# Preoperative Cyfra21-1 and SCC-Ag serum titers predict survival in patients with stage II esophageal squamous cell carcinoma

**DOI:** 10.1186/1479-5876-10-197

**Published:** 2012-09-21

**Authors:** Xun Cao, Lin Zhang, Gui-Rong Feng, Juan Yang, Ruo-Yan Wang, Jun Li, Xiao-Min Zheng, Yu-Jing Han

**Affiliations:** 1 State Key Laboratory of Oncology in South China, Cancer Center, Sun Yat-Sen University, No.651, Dongfeng Road East, 510060, Guangzhou, China; 2 Department of Thoracic Oncology, Guangzhou, China; 3 Clincal Laboratory, Guangzhou, China; 4 Department of Preclinical Medicines, Cancer Center, Sun Yat-Sen University, Guangzhou, China; 5 College of Traditional Chinese Medicines, Southern Medical University, Guangzhou, China

**Keywords:** Esophageal squamous cell carcinoma, Cyfra21-1, SCC-Ag, Prognosis

## Abstract

**Background:**

The purpose of this study is to evaluate the predictive significance of preoperative serum level of cytokeratin 19 fragments (Cyfra21-1) and squamous cell carcinoma antigen (SCC-Ag) after complete resection in patients with stage II esophageal squamous cell carcinoma (ESCC).

**Methods:**

Between 1995 and 2006, a total of 379 patients in stage II ESCC who underwent complete resection were consecutively recruited. Statistical analyses were applied to test the associations between preoperative serum titers of Cyfra21-1 and SCC-Ag, clinicopathological factors and prognoses.

**Results:**

Preoperative high and normal serum level of Cyfra21-1 and SCC-Ag were found in 47.8%, 52.2% and 72.8%, 27.2%, respectively. The 1-, 3-, 5-year overall survival rate for the entire cohort of patients was 95%, 78%, and 56%, respectively. Median overall survival (OS) was 45.3 months longer in patients with low preoperative serum level of Cyfra21-1 (91.9 months) than those with high preoperative serum level of Cyfra21-1 (46.6 months) (P < 0.001). Median OS among patients with SCC-Ag-low level was also longer than those with SCC-Ag-high level (89.7 vs. 63.7 months, P < 0.001), especially for those with stage IIB (P < 0.001). After multivariate analysis, along with pTNM stage, preoperative serum level of Cyfra21-1 and SCC-Ag were independently and significantly predictive factors (P < 0.001, P < 0.001). Furthermore, the five-year survival rate in double-low subset, either-low subset and double-high subset was 100%, 83% and 27%, respectively (P < 0.001).

**Conclusions:**

The preoperative serum level of Cyfra21-1 and SCC-Ag are independently significant predictors which negatively affected the survivals of patients with stage II ESCC.

## Background

Esophageal cancer is one of the least studied and deadliest cancers worldwide and the fourth most frequent cause of cancer-related deaths in China [1-3]. More than 90 percent of esophageal cancers are squamous cell carcinoma in China [3,4]. During the past four decades, important changes have occurred in the therapeutic patterns associated with this disease, such as surgery combined with chemotherapy and/or radiotherapy. Recent advances in diagnosis, staging and treatment of this neoplastic condition have led to promising and significant improvements in survival. Clinically, approximately 38-60% of patients present with stage II disease and the five-year survival ranging from 20% to 50% [5-8]. Additionally, to the best of our knowledge, despite having the same stage disease, most patients have different survival. Therefore, future studies should focus on the use of molecular biomarkers to predict patient survival and to select the patients who will benefit from specific treatment, including adjuvant chemotherapy.

Tumor-related proteins could secrete into the peripheral circulation of patients with cancer and may be detected by protein analysis. Peripheral serum tumor makers are non-invasive diagnostic tools for indentifying cancer, and are commonly used for the screening for cancer and as an indicator of treatment efficacy. Although cytokeratin 19 fragments (Cyfra21-1) and squamous cell carcinoma antigen (SCC-Ag) have been two of the most commonly used in the diagnosis of a variety of malignant tumor to date [9-14] the combination of them to predict the clinical outcomes of esophageal squamous cell carcinoma (ESCC) have not yet been well elucidated and established.

In the present study, we herein evaluated the associations between Cyfra21-1 level,

SCC-Ag level and clinicopathological parameters in a large cohort of ESCC patients with stage II. Furthermore, we analyzed the prognostic significance of preoperative serum level of Cyfra21-1 and SCC-Ag in these cases.

## Methods

### Ethics Statement

This study was approved by the medical ethics committee of Cancer Center at Sun Yat-Sen University.

### Patients

379 patients who underwent curative-intent surgery and with histologically proven stage II ESCC at Cancer Center, Sun Yat-Sen University between 1995 and 2006 were retrospective recruited. The cases exclude from the current study fulfilled the following criteria: (1) patients who had received preoperative treatment (chemotherapy and/or radiotherapy); (2) patients who presented with synchronous primary tumors and/or have previous malignant disease; (3) patients with a noncurative resection (R1); (4) patients who died of postoperative complications.

Preoperative evaluation included complete history and a physical examination, complete blood cell count, serum biochemistry, chest X-ray, esophageal barium meal, computed tomography scan of the chest and abdomen, endoscopy and an ultrasound scan of abdomen. The serum level of Cyfra21-1 and SCC-Ag were examined as a part of the routine preoperative evaluation within 1 week prior to surgery. According to the manufacturer (MODULAR ELECSYS E170, Roche, Mannheim, Germany; ARCHITECT SYSTEM i1000, Abbott, Tokyo, Japan), the normal upper limit was 3.30 ng/ml for Cyfra21-1 and 1.5 μg/L for SCC-Ag. According to normal upper limit of the tumor markers, the patients were divided into low and high subsets. The Cyfra21-1 and SCC-Ag levels were measured by a radioimmunoassay (RIA) in the clinical laboratory in our hospital. Stage was recorded based on American Joint Committee on Caner (2010) [15].

Adjuvant chemotherapy was performed in patients with stage IIB disease if the patients could tolerate additional treatment after curative-intent surgery, or unless the patients refused adjuvant chemotherapy. 206 patients with stage IIB received systemic chemotherapy (cisplatin-based combinations).

After completion of primary treatment, patients were followed up every 4–6 months in the first 3 years and every 12 months thereafter. The survival status was verified again using the best available methods in May 2010, including verifying the clinical attendance records and with direct telecommunication with the patient or their family.

### Statistical Analysis

The statistical analyses were performed using the SPSS 13.0 software package (SPSS, Inc., Chicago, IL). Overall survival (OS) was defined as the time from the date of surgery to the date of death or final clinical follow-up. The correlations between preoperative serum level of Cyfra21-1 and SCC-Ag and clinicopathological characteristics were assessed using the *χ*^2^ test. Multivariate Cox regression analysis was performed for all parameters that were found to be significant by the univariate analysis. Actuarial survival rates were plotted against time using the Kaplan-Meier method, and log-rank testing was used to compare the differences between the curves. *P* < 0.05 was considered statistically significant.

## Results

### Patient and Disease Characteristics

The median age of the patients was 60 years (range, 31 to 80 years), and 221 (58.3%) cases were men. Of 379 stage II ESCC patients, 253 (66.8%) received a left thoracotomy and 126 (33.2%) underwent an Ivor-Lewis/Three-field technique. The distribution pTNM stages was 127 stage IIA patients (33.5%), 252 stage IIB patients (66.5%). The patient and disease characteristics from all 379 patients with stage II ESCC are shown in Table 1. All further statistical analyses were performed on this population.

**Table 1:** Characteristics of the ESCC patients

**Characteristics**	**No. of patients (%) (*N*** **= 379)**
Age, years^†^	
≤ 60	128 (33.8%)
> 60	251 (66.2%)
Sex	
Male	221 (58.3%)
Female	158 (41.7%)
Operative procedure	
Left thoracotomy	253 (66.8%)
Ivor-Lewis/Three-field technique^‡^	126 (33.2%)
Tumor location	
Upper	20 (5.3%)
Middle	260 (68.6%)
Lower	99 (26.1%)
Tumor Grade	
Grade 1	80 (21.1%)
Grade 2	250 (65.9%)
Grade 3	49 (13.0%)
Pathological T status	
T1	39 (10.3%)
T2	213 (56.2%)
T3	127 (33.5%)
Pathological N status	
N0	127 (33.5%)
N1	252 (66.5%)
Pathological TNM stage	
Stage IIA	127 (33.5%)
Stage IIB	252 (66.5%)
Cyfra21-1 level	
Low (≤3.30 ng/ml)	198 (52.2%)
High (>3.30 ng/ml)	181 (47.8%)
SCC-Ag level	
Low (≤1.5 μg/L)	103 (27.2%)
High (>1.5 μg/L)	276 (72.8%)

Abbreviation: *T*, tumor; *N*, node; *TNM*, tumor-node-metastasis.

^†^ Median age; ^‡^Thoracic-abdominal-cervical incision.

### **The Preoperative Serum Level of Cyfra21-1 and**SCC-Ag**and Their Correlations With Clinicopathological Characteristics**

The preoperative serum levels of Cyfra21-1 and SCC-Ag were considered to be elevated as high when they exceeded 3.30 ng/ml and 1.5 μg/L, respectively. In the whole cohort, high and low serum level of Cyfra21-1 were in 181/379 (47.8%) and 198/379 (52.2%), respectively (Table 1). The preoperative SCC-Ag level was elevated in 72.8% (*n* = 276) of all patients with stage II ESCC (Table 1). Further analyses demonstrated that preoperative serum levels of SCC-Ag correlated closely with pN status (P = 0.005) and pTNM stage (P = 0.005), but no correlations were found between preoperative serum level of Cyfra21-1 and clinicopathological variables (*P* > 0.05, Table 2).

**Table 2:** The associations between Cyfra21-1 level, SCC-Ag level and the clinicopathological characteristics

	**No. of patients**	**Cyfra21-1 level**			**SCC-Aglevel**		
**Characteristics**	**(%) (*N*** **= 379)**	**Low**	**High**	*P *value^*^	**Low**	**High**	*P *value^*^
Tumor location				0.312			0.136
Upper	20 (05.3%)	12 (60.0%)	8 (40.0%)		9 (45.0%)	11 (55.0%)	
Middle	260 (68.6%)	129 (49.6%)	131 (50.4%)		71 (27.3%)	189 (72.7%)	
Lower	99 (26.1%)	57 (57.6%)	42 (42.4%)		23 (23.2%)	76 (76.8%)	
Tumor grade				0.191			0.771
Grade 1	80 (21.1%)	49 (61.2%)	31 (38.8%)		24 (30.0%)	56 (70.0%)	
Grade 2	250 (65.9%)	125 (50.0%)	125 (50.0%)		67 (26.8%)	183 (73.2%)	
Grade 3	49 (13.0%)	24 (49.0%)	25 (51.0%)		12 (24.5%)	37 (75.5%)	
pT status				0.899			0.594
pT1	39 (10.3%)	20 (51.3%)	19 (48.7%)		12 (30.8%)	27 (69.2%)	
pT2/3	213 (56.2%)	178 (52.4%)	162 (47.6%)		91 (26.8%)	249 (73.2%)	
pN status				0.059			0.005
pN0	127 (33.5%)	75 (59.1%)	52 (40.9%)		46 (36.2%)	81 (63.8%)	
pN1	252 (66.5%)	123 (48.8%)	129 (51.2%)		57 (22.6%)	195 (77.4%)	
pTNM stage				0.059			0.005
Stage IIA	127 (33.5%)	75 (59.1%)	52 (40.9%)		46 (36.2%)	81 (63.8%)	
Stage IIB	252 (66.5%)	123 (48.8%)	129 (51.2%)		57 (22.6%)	195 (77.4%)	

Abbreviation: *pT*, pathological tumor; *pN*, pathological node; *pTNM stage*, pathological tumor-node-metastasis stage.

^*^χ^2^ test.

### **The Relationships Between Preoperative Serum Level of Cyfra21-1 and**SCC-Ag**, Clinicopathological Characteristics and ESCC Patient Survival**

**Figure 1:**
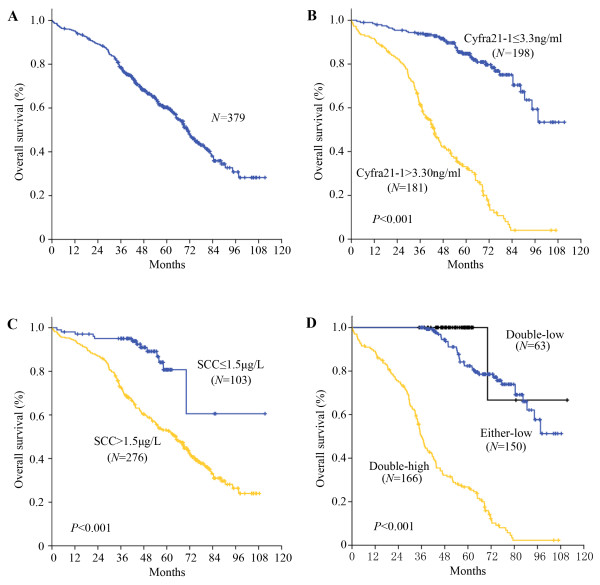
**Kaplan-Meier survival analysis in patients with stage II ESCC.** (**A**) Overall survival curve for whole cohort of patients with stage II ESCC; (**B**) Overall survival curve for whole cohort of patients with stage II ESCC according to the preoperative serum level of Cyfra21-1; (**C**) Overall survival curve for whole cohort of patients with stage II ESCC according to the preoperative serum level of SCC-Ag; (**D**) Overall survival curve for whole cohort of patients with stage II ESCC according to the preoperative serum level of Cyfra21-1 and SCC-Ag (double-normal subset vs. either-normal subset vs. double-high subset).

The median observation period was 52 months (range, 1.9 to 111.3 months), with 201 alive and 178 cancer-related deaths at the last clinical follow-up. The median and mean OS were 70.9 and 69.6 months, respectively. The 1-, 3-, 5-year overall survival rate for the entire cohort of patients was 95%, 78%, and 56%, respectively (Figure 1A).

In the Kaplan-Meier analysis, the preoperative serum level of Cyfra21-1 and SCC-Ag were closely associated with OS. For the whole cohort, median OS was 45.3 months longer in patient with low preoperative serum level of Cyfra21-1 (91.9 months) than those with high preoperative serum level of Cyfra21-1 (46.6 months) (*P* < 0.001, Table 3, Figure 1B). Median OS among patients with SCC-Ag-low level was also longer than those with SCC-Ag-high level (89.7 vs. 63.7 months, *P* < 0.001, Table 3, Figure 1C). Because the preoperative serum level of Cyfra21-1 and SCC-Ag was found to be significantly predictive factors for OS, we have further examined the relationship between preoperative serum level of Cyfra21-1 and SCC-Ag and survival based upon patient’s pTNM stage. This analysis showed that the preoperative serum level of Cyfra21-1 could distinguish OS when stratified by stage IIA and stage IIB (*P* < 0.001, *P* < 0.001, Table 3). On the other hand, in patients with stage IIB ESCC, the 5-year survival rate in SCC-Ag low group was significantly better (78%) than those of patients in SCC-Ag high group (45%) (*P* < 0.001, Table 3). However, no significant difference was observed in the 5-year survival rate in the patients with stage IIA disease between the patients with low SCC-Ag serum level and those with high SCC-Ag serum level (86% vs. 72%, *P* = 0.069, Table 3). The 5-year survival rate in double-low subset, either-low subset and double-high subset was 100%, 83% and 27%, respectively (*P* < 0.001, Figure 1D).

**Table 3:** Prognostic value of preoperative Cyfra21-1 and SCC-Ag level in ESCC patients

**Biomarker**	**Cyfra21-1**				**SCC-Ag**			
**Level**	**No.**	**Median OS**	**5-year survival**	*P *value^*^	**No.**	**Median OS**	**5-year survival**	*P *value^*^
Total				<0.001				<0.001
Low	198	91.9	85%		103	89.7	82%	
High	181	46.6	33%		276	63.7	53%	
pTNM stage								
Stage IIA				<0.001				0.069
Low	75	102.6	93%		46	85.7	86%	
High	52	59.9	54%		81	78.2	72%	
Stage IIB				<0.001				<0.001
Low	123	84.4	80%		57	73.7	78%	
High	129	41.1	25%		195	57.1	45%	

Abbreviation: *pTNM stage*, pathological tumor-node-metastasis stage.

^*^ Log-rank test.

Several clinical and pathological factors were found to be associated with stage II ESCC patient survival on both univariate and multivariate analyses (Table 4). On multivariate analysis, stage IIB (HR, 2.191; *P* < 0.001), Cyfra21-1 > 3.30 ng/ml (HR, 7.149; *P* < 0.001), and SCC-Ag > 1.5 μg/L (HR, 2.926; *P* < 0.001) negatively affects survival of surgically resected stage II ESCC patients.

**Table 4:** Univariate and multivariate Cox regression analysis for overall survival in patients with ESCC

	**Univariate analysis**			**Multivariate analysis**		
**Characteristics**	**HR**	**95% CI**	*P *value^*^	**HR**	**95% CI**	*P *value^*^
Age, years	1.014	1.001 to 1.028	0.038	0.998	0.985-1.012	0.764
Sex						
Male vs. Female	1.212	0.902 to 1.629	0.202	-	-	-
Tumor location						
Upper vs. Middle vs. Lower	0.980	0.558 to 1,611	0.318	-	-	-
Tumor Grade						
Grade 1 vs. 2 vs. 3	1.668	1.085 to 2.166	0.056	-	-	-
pTNM stage						
Stage IIA vs. Stage IIB	2.283	1.600 to 3.257	<0.001	2.191	1.533 to 3.132	<0.001
Cyfra21-1 level						
Low vs. High	7.439	5.178 to 10.686	<0.001	7.149	4.961 to 10.303	<0.001
SCC-Ag level						
Low vs. High	3.940	2.229 to 6.964	<0.001	2.926	1.655 to 5.175	<0.001

Abbreviation: *HR*, hazard ratio; 95% CI, 95% confidence interval; *pTNM stage*, pathological tumor-node-metastasis stage.

^*^ Cox proportional hazards model.

## Discussion

A complete surgical resection is considered to be the first line treatment for individuals with localized ESCC [3,16,17]. However, to the best of our knowledge, substantial differences in survival were observed for the same TNM stage patients. After complete surgical removal of the tumor, the five-year survival rate is 30 to 40 percent for stage IIA disease, and 10 to 30 percent for stage IIB disease [5-8]. Those phenomena indicate that stage II patients who undergo surgery form a heterogeneous group in which the disease progression and survival rate can vary considerately. Thus, there is a significant need for early identification of patients who are at increased risk of disease progression after their primary treatment. A blood-based biomarker is attractive filter because blood is easily accessible and measurements may be repeated over time [18-20].

Several previous studies have revealed that the significance of Cyfra21-1 and SCC-Ag level for cancer screening [21-23]. However, the predictive analysis of Cyfra21-1 and SCC-Ag level in ESCC, especially in stage II ESCC, has not been well elucidated and studied. Based on unique etiology, patient characteristics, uniform treatment modalities and long term follow-up, the current study is the first to systematically evaluate the prognostic value of preoperative serum level of Cyfra21-1 and SCC-Ag in ESCC. Our findings indicate that preoperative Cyfra21-1 and SCC-Ag level significantly associated with survival in patients with stage II ESCC.

Cyfra21-1 is a member of the keratin family. It is a protein that in humans is encoded by the *KRT19* gene [24,25]. Its expression can be observed in many normal tissues, and it is overexpressed in lung cancer, colorectal cancer and breast cancer [26-29]. SCC-Ag is a sub-fraction of the tumor antigen TA-4, a 48 kDa glycoprotein first isolated by Kato and Torigoe [30]. SCC-Ag is released and often elevated in patients who have squamous cell carcinomas. SCC-Ag has become a promising aid for the management of squamous cell carcinoma in a variety of sites, such as cervical cancer, head and neck cancer and esophageal cancer. The previous studied have indicated that Cyfra21-1 and SCC-Ag are sensitive tumors marker in malignant disease, particularly in squamous cell type [19,31,32]. In the present study, our results firstly showed a significant correlation between the preoperative serum level of SCC-Ag and clinicopathological factors (pN status, *P* = 0.005; pTNM stage, *P* = 0.005). This finding corresponds with the studies from France and Poland [19,21]. However, we failed to reveal any correlation between the preoperative serum level of Cyfra21-1 and patient disease characteristics. In contrast, Moro et al. and Hanagirl et al. reported that preoperative Cyfra21-1 was associated with tumor stage in non small cell lung cancer [21,33]. We believe this result can be explained by three particular factors. Firstly, various cutoff points might lead to various results. Secondly, the heterogeneity of tumors might result in the discrepancies in the findings between the previous literatures and our study. In addition, cohort-specific differences such as ethnicity and epidemiology might also explain the observed phenomena. However, this conclusion merits additional research.

To date, the use of serum markers has not been accepted in assessing prognosis or assigning treatment after complete resection for ESCC. The prognostic values of Cyfra21-1 and SCC-Ag were also still controversial. Cyfra21-1 is a small molecular protein responsible for structural integrity of epithelial cells, which also functions to promote cell growth, adhesion, migration and invasion [34]. High Cyfra21-1 serum titers have been demonstrated to correspond to poor prognosis in patients with non small cell lung cancer and colorectal cancer [21,26,33]. SCC-Ag may be involved in the malignant behavior of squamous cell cancers, functioning in invasion and/or metastasis. Associations between serum SCC-Ag concentrations and patient survival have also been observed [19,33,35,36]. In the current study, when we focused exclusively on the survivals of patients, along with the pTNM stage, the preoperative serum level of Cyfra21-1 and SCC-Ag were shown to be independent and significant prognostic parameter in surgically resected stage II ESCCs (Table 4). Interestingly, in the subset analyses, Cyfra21-1 and SCC-Ag level displayed statistically significant effect on survival in stage IIB patients. Furthermore, the present study indicated that the double low subset, either-low subset and double-high subset have significantly different survival (*P* < 0.001, Figure 1D), suggesting that prognostic information may be attainted from initial serum titers of Cyfra21-1 and SCC-Ag.

Although this study has revealed the prognostic significance of preoperative Cyfra21-1 and SCC-Ag serum titers, we acknowledged that there were some limitations in our study. Our study is a retrospective study, relied exclusively on a single-institutional database. Additional investigation into this area must be performed.

## Conclusions

In conclusion, our findings identified that stage II ESCC patients with poor prognoses are certainly predictable. The high preoperative serum level of Cyfra21-1 and SCC-Ag are independently significant predictors which negatively affected the survivals of patients with stage II ESCC. A future confirmatory study or investigation for patient-tailored adjuvant treatment with stratification according to Cyfra21-1 and SCC-Ag serum level are therefore warranted to evaluate whether such selection may ultimately improve patient prognosis after surgery for this deadly cancer.

## Competing interests

The authors declare that they have no competing interests.

## Authors’ contributions

XC, LZ performed the statistical analysis, drafted the manuscript and participated in the sequence alignment. GRF participated in the design of the study and participated in the sequence alignment. JY participated in the sequence alignment. RYW, JL and XMZ carried out data acquisition. YJH conceived of the study, and participated in its design and coordination and helped to draft the manuscript. All authors read and approved the final manuscript.
